# Orientation and Influence of Anisotropic Nanoparticles in Electroconductive Thermoplastic Composites: A Micromechanical Approach

**DOI:** 10.3390/polym17243273

**Published:** 2025-12-09

**Authors:** Lisa Windisch, Björn Düsenberg, Maximilian Nowka, Karl Hilbig, Thomas Vietor, Carsten Schilde

**Affiliations:** 1 Institute for Particle Technology, Technische Universität Braunschweig, Volkmaroder Str. 5, 38104 Braunschweig, Germanyc.schilde@tu-braunschweig.de (C.S.); 2 Institute for Engineering Design, Technische Universität Braunschweig, Hermann-Blenk-Str. 42, 38108 Braunschweig, Germany; m.nowka@tu-braunschweig.de (M.N.); k.hilbig@tu-braunschweig.de (K.H.); t.vietor@tu-braunschweig.de (T.V.)

**Keywords:** material extrusion, electrical conductivity, polymer composite, fused filament fabrication, nanoindentation, particle distribution

## Abstract

The integration of electrically conductive functionalities into polymer components via additive manufacturing has gained increasing relevance across fields such as sensing, energy storage, and structural electronics. Achieving reliable performance in such applications requires a deeper understanding of how processing conditions affect the internal structure of conductive thermoplastic composites—particularly the orientation and distribution of anisotropic fillers. This study analyzes a PLA-based composite containing carbon nanotubes, carbon black, and graphite flakes to evaluate the influence of extrusion temperature on electrical resistivity and micromechanical properties. To complement scanning electron microscopy, a novel micromechanical mapping approach based on nanoindentation was applied, enabling spatially resolved analysis of local stiffness and hardness. Results show that increasing extrusion temperature improves filler dispersion and alignment, enhancing conductivity and mechanical homogeneity—up to a threshold of 210 °C. Even small temperature changes significantly affect particle orientation and distribution. Unlike global resistivity measurements, the combined use of nanoindentation and microscopic imaging reveals location-specific structural phenomena and filler behavior within the matrix. This newly established method provides high-resolution insight into internal composite architecture and offers a robust foundation for optimizing process-structure-property relationships in conductive polymer systems.

## 1. Introduction

Additive manufacturing (AM) has emerged as a transformative technology, enabling the production of complex geometries and functional components across a variety of fields, from aerospace to medical devices. Of particular interest is Material Extrusion of polymers (MEX), which is an umbrella term that combines both printing objects from a spool of filament (Fused Filament Fabrication; FFF) or polymer granulate (Fused Granulate Fabrication; FGF). One of the most significant advancements in this domain is the development of electrically conductive composites, which facilitate the integration of sensors [1,2,3,4,5,6,7,8,9,10,11], actuators [3,12,13,14], electrical connections [15,16,17,18,19,20], energy storage systems [18], and other electronic functionalities [18,21] directly into additively manufactured structures.

The properties of these electrically functional components are primarily influenced by the choice of materials [3,18,22,23,24], filament manufacturing process parameters [10,25,26,27,28], and process parameters set during Hot Melt Extrusion (HME) [3,9,17,23,29,30,31,32]. Various parameters impact the interaction between the conductive additives and the electrical insulating polymer matrix, which directly affects the electrical resistivity of the resulting structures [23,33,34,35,36,37,38,39]. Studies have shown that the process parameters during filament extrusion, particularly the temperature profile and screw speed, play a crucial role in determining the specific resistivity of the filament [14]. Once the filament is extruded, subsequent FFF process parameters can maintain or slightly improve the resistivity but are unable to reduce it further.

Gonçalves et al. [40] demonstrated that varying screw geometry could reduce specific resistance, while Paz et al. [41] showed that increases in both temperature and screw speed lead to improved conductivity. These relationships were also observed in prior works of the authors, with the temperature profile being the dominant factor [14].

So far, electrical resistivity has been primarily investigated using two methods: two-wire [3,17,31] and four-wire resistivity measurements [12,14,19,23,29,30]. However, these methods can only provide information on the conductivity of the entire test specimen. The electrical properties of polymer-particle composites largely depend on the internal structure of the material. Even small changes in factors such as particle size, distribution, and morphology, polymer structure, or dispersion quality can have a significant impact on the specific resistance of the material, to the extent that even small local irregularities can interfere with the functional integrity of an entire structure [42].

Due to this sensitivity, numerous approaches have already been studied to clarify the particle orientation and alignment [43] in the polymer melt or in the solidified polymer. These methods can be divided into three categories: Process methods, ultrasonic methods, and variations in established laboratory methods [44].

In- and online-based process methods offer the advantage of being able to measure almost precisely what is happening in the process at a specific point in time. As extrusion processes often take place extremely quickly, only split seconds are available for measurement. Present studies have mainly focused on the installation of a bypass fitted with analytical equipment (e.g., Raman, Near-Infrared (NIR), and ultrasonic (US) sensors) after the extruder nozzle (round hole or slit die) [45,46,47]. In all cases, microscopic images were taken to verify the spectroscopic observations. However, these methods are only suitable for translucent melts with mass concentrations of up to 1 weight-%. A promising approach was presented by Wöckel et al. [44] by utilizing acoustic transmission of polymer melts to gather information on particle size, distribution, and mass content. This method can also be applied to highly concentrated, opaque material systems without dilution. However, the establishment of the technique is very complex and difficult to apply to frequently changing material systems, as occurs in research and development.

Classic laboratory methods are not affected by the mass content limitations. They usually take place with a time delay and are performed after the process. Optical methods are widely used, especially scanning electron microscopy (SEM). For example, Yoon et al. [48] investigated the distribution of carbon nanotube (CNT) particles in a polymer to improve the electric heating capability and heat-induced electrical stability. Huang et al. [49] demonstrated how the process time affects the localization of different CNT types in a PVDF/ABS (polyvinylidene fluoride/acrylonitrile butadiene styrene) matrix and thus influences the dielectric properties. The merely global observations obtained from the spectroscopic analyses could be quantitatively verified with SEM images. Zhang et al. [50] revealed the complex nanostructure of CNC/GO (cationic cellulose nanocrystals/graphene oxide) filaments using SEM.

Furthermore, mechanical properties can be used to conclude the distribution and orientation of the particles in the composite [51,52,53]. In the area of plastics, impact and tensile strength are often considered. Most studies were conducted with fiber-based fillers such as wood or nylon [54,55], but also for carbon-based materials such as carbon fibers [56]. Similar to electrical conductivity, a disadvantage of such methods is that the properties can only be determined globally for the whole test specimen. A local characterization of the particle network is therefore not possible.

None of the methods discussed so far can unravel the internal structure of highly filled polymer composites with nano- and microparticulate, anisotropic fillers. Particularly, not at any given sample location, to be able to analyze local phenomena such as edge effects.

However, in the field of micromechanics, local variations in mechanical properties are the key interest and have been the subject of many studies in recent years [57,58,59,60]. So far, micromechanical analyses are barely used in the field of polymer composites, especially for those intended for additive manufacturing. Using a micromechanical approach, which has yet to be widely applied to electrically conductive composites, this study aims to bridge the gap between electrical performance and internal composite structure.

This study aims to address this gap by investigating how the orientation and distribution of anisotropic, nano- and microscale fillers in solidified thermoplastics can be quantitatively assessed. This approach will allow for more accurate conclusions to be drawn about filler distribution and orientation, leading to potential optimizations in conductivity.

## 2. Materials and Methods

### 2.1. Material

**Figure 1:**
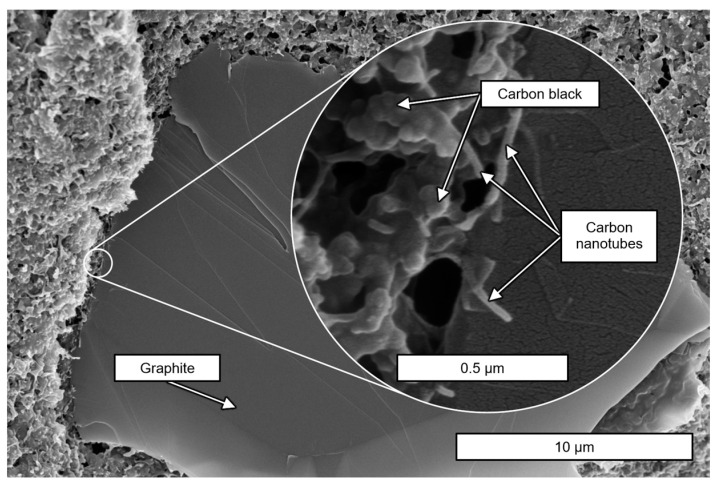
SEM image of the cryo-fracture surface perpendicular to the extrusion direction of commercial AlfaOhm filament.

For this study, the commercially available thermoplastic composite material AlfaOhm (FiloAlfa, Milan, Italy) was used. The supplier provides it as filament or pellets, whereby the granules were used as feedstock for the single screw melt extrusion (SSE) process in the present study. Granules are spherical or oval-shaped, with a mean diameter of 2.5 to 4 mm. The general public material description of AlfaOhm specifies it as a PLA/CNT blend. Being a trade secret, the precise composition is unknown. Contreras-Naranjo et al. [61] further specified the PLA/CNT material and reported that it contains carbon black (CB) as well as approx. 3 weight-% of multiwalled CNTs with dimensions of 8 ± 3 nm in diameter, which are synthesized by catalytic chemical vapor deposition (CCVD). Their experimental procedure revealed a particulate mass content of 34.55% with an estimated 0.1 CNT/CB ratio. Figure 1 shows a scanning electron microscope (SEM) image of the round front end (perpendicular to the extrusion flow) of the material (filament as provided by the supplier). Alongside the fillers previously discussed, graphite (Gr) flakes could be detected throughout the entire material. AlfaOhm is therefore evidently a PLA/CNT/CB/Gr blend.

The manufacturer recommends an extrusion temperature between 190 and 210 °C. There are no separate specifications for the processing temperatures of feedstock in granular form.

### 2.2. Material Extrusion and Filament Production

The filament is produced from granules using a laboratory-scale filament production system developed in-house. It consists of a single-screw extruder, two water baths for cooling the filament strand, an automatic diameter measuring unit, and a module for winding the filament strand onto spools.

The single screw extruder has a screw diameter of 20 mm and a length-to-diameter ratio of *L*/*D* of 14.5. The extruder is equipped with four heating zones. The die orifice is round with a diameter of 2.0 mm. The nominal filament diameter of 1.75 mm is set by the haul-off speed. In this study, the filament is produced at a constant screw speed of 19.8 rpm with variable temperature profiles. The linear temperature profile is derived from the temperature in the pellet feed zone (22 °C, room temperature) and the melt ejection zone (variable). For parameter variation, the temperature in the ejection zone is increased in 10 °C steps from 180 to 220 °C. This covers the recommended processing temperature range (190–210 °C) and includes one data point above and one data point below. For example, for a melt ejection zone temperature of 180 °C, the temperature profile would be as follows: 180 °C (100%)/135 °C (75%)/90 °C (50%)/45 °C (25%)/22 °C (room temperature). All temperature profiles are summarized in Table 1.

Immediately after exiting the nozzle, the polymer melt enters a hot water basin at 80 °C and solidifies. Due to the similar densities of water and PLA, solidification occurs almost without the reduced influence of gravity. After the hot water bath, the filament is cooled to room temperature in a cold-water bath (22 °C). The filament is then rolled onto spools.

Each specimen represents a parameter set and is referred to as #1–#5 in the further course of this study. Where extrusion temperatures are stated, only the highest temperature at the nozzle (180–230 °C) is mentioned (see Table 1).

### 2.3. Sample Preparation

**Figure 2:**
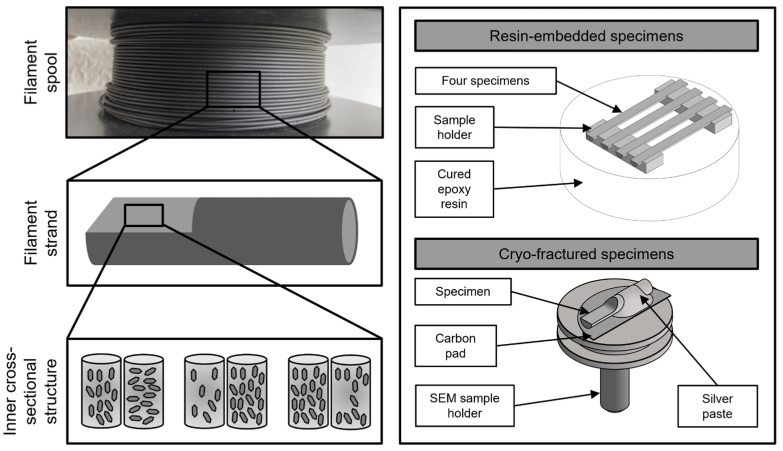
Description and objective of the sample preparation steps (**left**) and schematic drawings of the cryo-fractured and resin-embedded samples (**right**). For the electron microscopic examination, the resin-embedded specimens are also attached to a pin stub with a carbon pad and contacted with conductive colloidal silver paste on the end faces of the filament pieces.

In order to evaluate the orientation and distribution of the particulate fillers in the solidified PLA/CNT/CB/Gr composite, the inner structure of the filaments must be analyzed. For this purpose, small pieces of the circular, cylindrical filament pieces (length = approx. 1–2 cm; diameter = 1.75 mm) are prepared to expose the cross-sectional area in the direction of the extrusion flow. By exposing the inside of the sample near the largest diameter, effects from the edge to the innermost center can be investigated. For this, two techniques are used: cryogenic fracturing and embedding and polishing in epoxy resin. A schematic illustration of both sample preparation outcomes is illustrated in Figure 2.

The first technique involves cryogenically cooling small filament strands with liquid nitrogen. A side cutter is used to initiate a brittle fracture from the end face. Many potential specimens are produced as the propagation of the fracture edge cannot be controlled. The specimen with the most central and parallel fracture surface is selected. The filament piece is fixed to a carbon pad with conductive colloidal silver paste (EMS #12640, Electron Microscopy Sciences, Hatfield, PA, USA).

To statistically analyze the micromechanical properties using nanoindentation, the specimens’ surface roughness must be brought down to a minimum. For this, the samples were embedded in resin, sanded to the center of the filament, and subsequently polished. For each sample, four filament strands were fixed next to each other in a round cylindrical embedding mold (diameter = 25 mm). EpoFix Resin and EpoFix Hardener (Struers GmbH, Willich, Germany) were used as the cold embedding system. To protect the samples from porosity, cracks, and fissures during curing, they were stabilized for 60 min at 0,11 bar using vacuum impregnation (CitoVac, Struers GmbH, Willich, Germany). After a curing time of approx. 24 h, the embedded specimens were processed in several steps using an automatic, microprocessor-controlled grinding/polishing machine (Tegramin-30, Struers GmbH, Willich, Germany). Silicon carbide (SiC) abrasive paper with a grain size of 500, 1000, and 2000 was used for coarse grinding. The sanding times varied depending on the sample height. The exposed inner surface of the filament pieces was then fine-ground with a 9 µm (DiaPro Allegro/Largo 9 µm on a MD-Largo grinding surface, Struers GmbH) and a 1/4 µm diamond suspension (DiaPro Nap 1/4 µm on a MD-Nap polishing cloth, Struers GmbH).

In order to summarize which sample type is investigated using which of the analytical methods discussed in the following chapters, Table 2 provides an overview.

### 2.4. FT-IR and TGA

Fourier transform infrared spectra (FT-IR) were recorded using a VERTEX 70v (Bruker, Ettlingen, Germany) with a spectral range of 4000–400 cm^−1^. For sample preparation, thin pinhead-sized samples were cut from the filaments by hand using a scalpel. Due to the high particulate mass content and the associated elevated absorption capacity of the filaments, a triple determination was carried out. Thermogravimetric analysis (TGA; TGA/DSC 3, Mettler Toledo GmbH, Gießen, Germany) was carried out under both oxygen and nitrogen atmospheres. The samples were heated from 30 to 950 °C at a heating rate of 10 °C/min.

### 2.5. Electrical Resistivity of Filament Specimens

**Figure 3:**
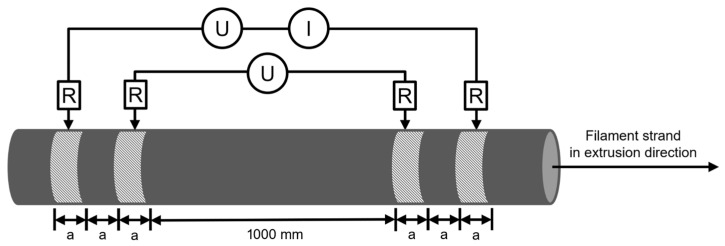
Four-wire test setup. The cross-hatched surfaces are coated with conductive colloidal silver paste. *U* = voltage, *I* = current and *R* = resistance. The distance between the contacts is defined as *a* = 10 mm.

The resistivity ρ of the extruded filament was measured based on the DIN EN ISO 3915:2022-5 standard. Resistivity is a geometry-independent measure and is the inverse of electrical conductivity. However, this standard cannot be directly applied to the measurement of cylindrical polymer-particle composite filaments with anisotropic electrical properties. Therefore, the measurement procedure was adapted. For this study, five 1200 mm long filament pieces were cut from a roll of filament of each batch. Each batch represents one parameter set (see Table 1). A Kelvin (four-wire) measurement configuration was employed to eliminate lead resistance effects, in contrast to the conventional two-wire method. This configuration also mitigated contact resistance. However, using a conductive contact aid further improved measurement repeatability. Accordingly, four annular contacts, each 10 mm wide, were applied to the filament. For this, conductive colloidal silver paste was used (EMS #12640; Electron Microscopy Sciences, Hatfield, PA, USA). At each end of the filament strand, there was a contact for force and sense, as schematically shown in Figure 3. The two sense contact points were placed 1000 mm apart, while the distance between the force and sense contacts was *a* = 10 mm.

The conductive colloidal silver paste was left to dry for a minimum of 24 h. The four-wire resistance measurement was conducted using a Keithley 2460 source meter (Keithley Instruments, Solon, OH, USA) at a measuring current of 100 µA. Due to the samples’ high conductivity, a voltage of 5 V was not exceeded, thereby reducing the impact of self-heating due to thermal power loss. This eliminated any potential influence on the test results due to sample heating. The resistance measurement was conducted at a constant temperature of 22 °C. To calculate the specific resistance, it was necessary to determine the average electrically conductive cross-sectional area. This was achieved by measuring the diameter at three points (see Figure 3) with a micrometer screw (QuantuMike 293-140-30, Mitutoyo Corporation, Kawasaki, Japan).

### 2.6. Microstructural Analysis: Methodological Approach to Reveal Particle Orientation and Distribution in the Composite

#### 2.6.1. Imaging Methods

Images of the cryo-fractured and polished samples’ cross-sections were taken with a scanning electron microscope (SEM; Helios NanoLab G3 UC, FEI Deutschland GmbH, Dreieich, Germany) in secondary electron mode (acceleration voltage set to 3 and 5 kV). The samples were placed on carbon pads and coated with a 4 nm Pt coating using a sputter coater (EM ACE600, Leica Microsystems, Wetzlar, Germany). To improve the electrical conductivity between the sample and the holder, the specimens were coated with silver conductive grease.

In order to gain insight into the particle distribution across the entire diameter of the filaments, images were taken with a confocal Raman microscope (alpha300 R; WITec GmbH, Ulm, Germany). For this purpose, it was used as an optical microscope without the laser function. The images were taken with a 50× DIC objective (EC EPIPLAN Neofluar, Zeiss, Oberkochen, Germany).

#### 2.6.2. Micromechanical Surface Properties

The micromechanical surface properties of the polished composite samples were obtained via nanoindentation (Hysitron TI 950 TriboIndenter, Bruker, Ettlingen, Germany) equipped with a diamond Berkovich tip. The tip geometry is a self-similar triangular pyramid with a total included angle of 142.3°. In order to combine statistical relevance with reasonable measurement times, square grids of 625 (25 × 25) indents were created. The measuring time per pattern was approx. 22 h. According to Arfsten et al. [62] and Barth et al. [63], 40 indents per nanoindentation depth provide sufficient statistical certainty in order to conclude the distribution of mechanical properties. The single indents in the grid were applied to reach a maximum depth of 250 nm, and they had an inter-indent spacing of 5 µm. Due to the low forces, in very few cases, the measurement of an indent in a pattern failed [64]. The measured value of this particular indent was then calculated as the average of the surrounding indents.

**Figure 4:**
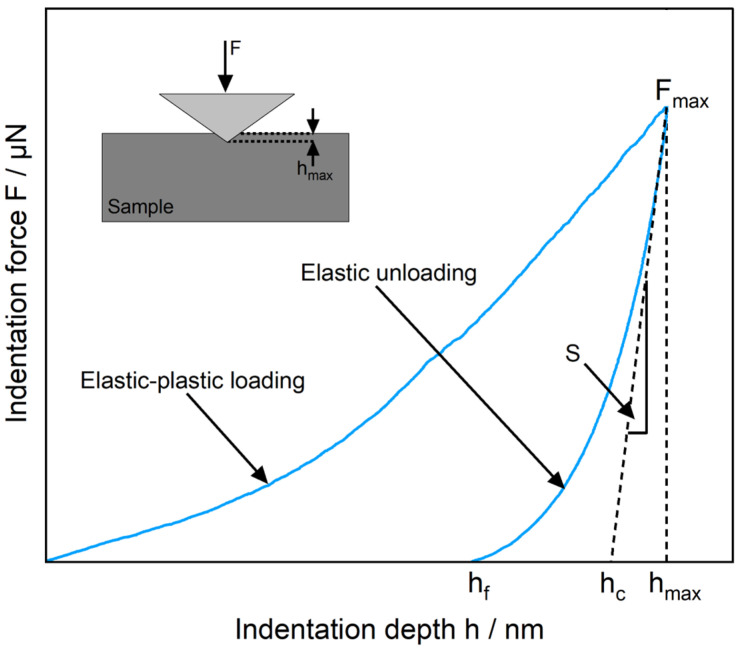
Schematic representation of an indentation load–load-displacement curve.

The two most important mechanical parameters that are determined with nanoindentation are the hardness (*H*) and the elastic modulus (Young’s modulus; *E*), which are derived from the method according to Oliver and Pharr [65,66]. To illustrate the measurement principle, the schematic relationship between indentation load (*P*) and displacement (*h*) for a full cycle of loading and unloading is plotted in Figure 4. The most important values were the peak load *P_max_*, the maximum indentation depth *h_max_*, the remaining indentation depth after the unloading step *h_f_*, and the slope of the unloading curve *S*, which described the contact stiffness of the material. This provided the fundamental relationships for calculating *H*:

(1) $$H\;=\;\frac{P}{A}$$

where *P* is the load and *A* is the projected contact area, and *E*:

(2) $$E_{r}\;=\;\frac{\sqrt{\pi }}{2ß}\frac{S}{\sqrt{A}}$$

where *E_r_* represents the reduced elastic modulus and ß is a geometric constant. For the Berkovich triangular pyramidal indenter, a *ß* value of 1.034 is usually assumed. *E_r_* is used to consider not only the elastic deformation of the test material but also that of the indenter tip.

## 3. Results and Discussion

### 3.1. Influence of the Extrusion Temperature Profile on the Electrical Resistivity

**Figure 5:**
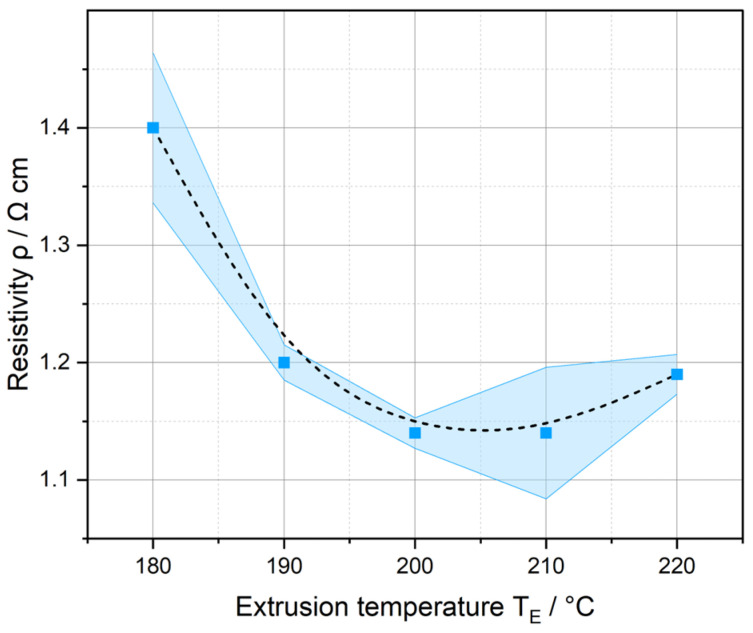
Resistivity of the filaments as a function of the process temperature during single screw Hot Melt Extrusion.

The resistivity *ρ* as a function of the extrusion temperature *T_E_* is plotted in Figure 5. The band around the points represents the standard deviations. As *T_E_* rises, a drop in resistance can be observed. From 200 to 210 °C, however, it increases again. The reasons for this can be found in the rheological behavior of the polymer melt. If the melt is too viscous (*T_E_* is too low), localized accumulation and depletion of the fillers in the PLA/CNT/CB/Gr composite can occur [67]. With rising *T_E_*, the viscosity of the melt decreases, whereby the particles are distributed more evenly, and structures such as agglomerates or aggregates can disintegrate more effectively. This results in a more homogeneous particulate network with fewer interruptions in the electrical conduction paths, which improves conductivity. Polymers are temperature-sensitive, which is why structural change due to thermal stress sets in at material-specific temperatures, leading to degradation at a certain point. The onset of structural change is one possible reason why the measured *ρ* increases again above 210 °C.

This results in a parabolic relationship, which is plotted as a B-spline in Figure 5. The electrical conductivity, therefore, allows initial conclusions to be drawn about the material behavior in the measured area (10 cm long filament section). However, it only serves as a qualitative measured variable, as the material system is viewed as a black box. The distribution and three-dimensional orientation (in the case of anisotropic particles such as graphite and carbon nanotubes) of the fillers have a significant effect on the conductive paths. No data-based conclusions can be drawn about the particulate network using this measurement method.

### 3.2. Microscopic Investigation of the Internal Composite Structure

**Figure 6:**
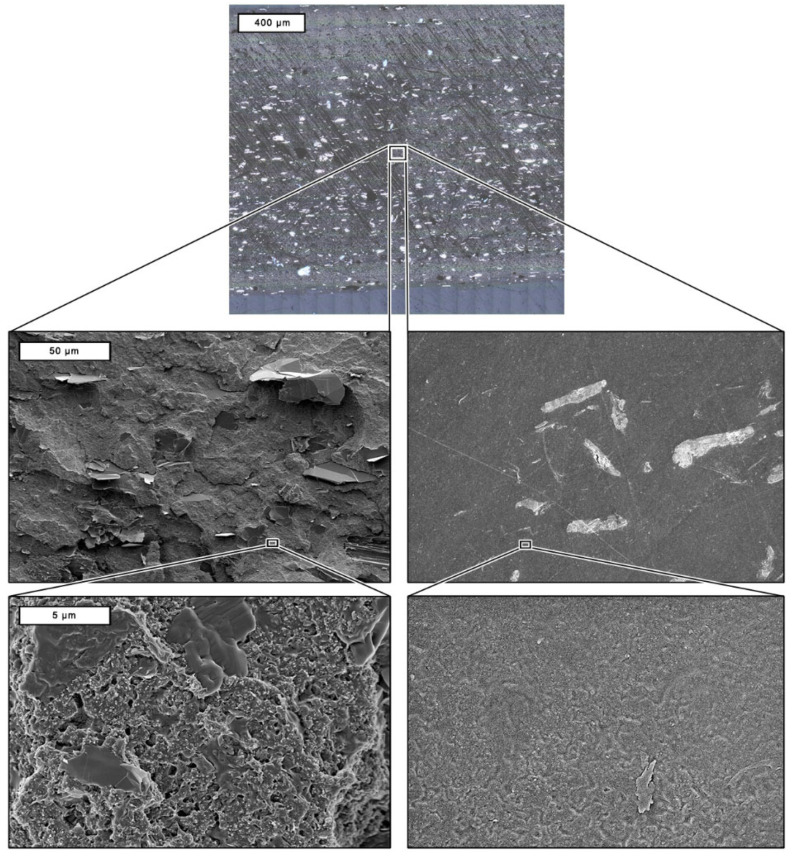
Comparison of the structures that can be resolved with the respective optical analysis methods used in this study.

Optical analysis techniques are commonly used to elucidate the particle network in solidified thermoplastic composites. This allows to draw initial conclusions on the influence of the particle network in the composite on the electrical conductivity. Different phenomena in the composite can be observed using multiple microscopic methods and magnifications. Therefore, the results of the imaging methods and sample preparations used for this study are shown in Figure 6 using sample #5 as an example.

The light microscope image in the first row covers the total cross-section of the filament segment (1.75 cm) of a resin-embedded sample. This method is suitable for obtaining a quick overview of the specimen and the distribution homogeneity of the coarser (two-digit µm range) fillers. Such carbon-based particles and agglomerates can be easily recognized as whitish structures due to backscattering effects. The dark horizontal stripes are related to the grinding and polishing process. The overview shows that particle depletion (especially of coarser particles such as graphite) occurs towards the edge of the cylindrical filament [46,47]. This is a well-known polymer-filler interaction that is caused by the different thermal expansion coefficients of carbon allotropes and the polymer and is described as a vulcanization skin, particularly in the field of elastomers. When the hot polymer melt emerges from the extruder nozzle, the polymer expands significantly more than the fillers and covers them with a thin layer before the material solidifies [68].

The rows below show electron microscope (SEM) images of the cryo-fractured (left column) and resin-embedded (right column) samples. The former appears much more structured and contrasty in comparison, which is caused by manually breaking the sample in the preparation process. Graphite particles (Gr) are present as coarse, planar, and clearly visible structures. However, the distribution of the carbon black (CB), the carbon nanotubes (CNTs), and their interaction with the PLA matrix can only be resolved at a higher magnification. The images of the polished samples lose contrast when viewed by SEM due to the sample preparation. An important factor for the SEM image quality is the electrical conductivity of the specimens. The large sample volume and the lack of direct contact between the filament strand and the carbon pad (see schematic drawing in Figure 2 on the right) complicate image generation as the epoxy resin block enclosing the samples has an electrically insulating effect. Here, the fillers can be easily detected due to their higher density compared to PLA, as they appear as backscattered electrons in a whiteish color. In contrast to the cryo-fractured samples, the dimensions of the Gr particles can be determined, and even smaller particle structures (e.g., the CB network) can be clearly identified as such.

**Figure 7:**
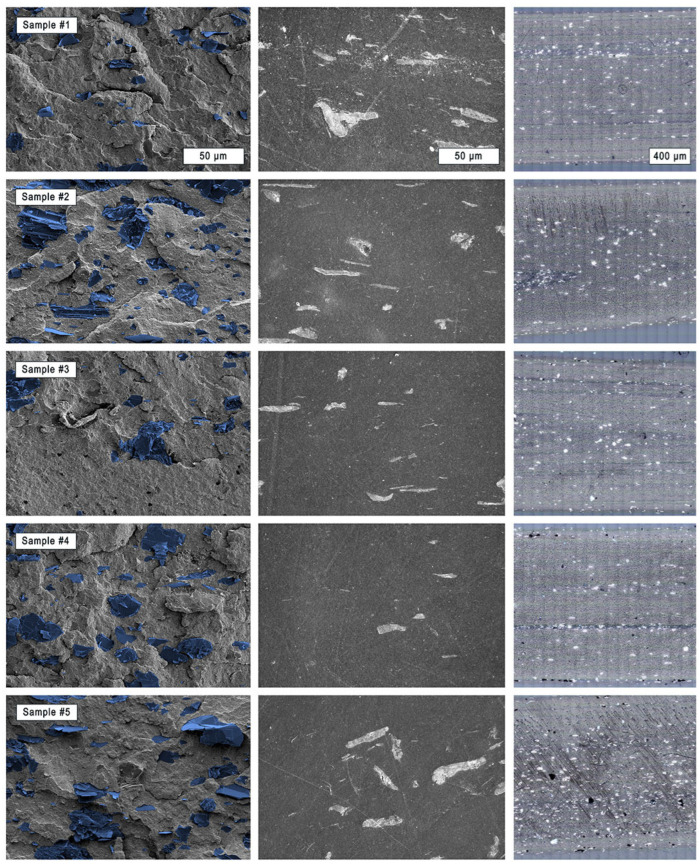
SEM images of cryo-fractured (**left**), polished (**middle**), and light microscopic images of the polished (**right**) specimens. The image acquisition location is in the concentric center of every sample. All three pictures in a row belong to one sample associated with one extrusion temperature profile.

Figure 7 compares SEM and light microscope images of the cryo-fractured and polished horizontal surfaces of the five samples processed at different extrusion temperatures. As already shown in Figure 6, the combination of different imaging methods and magnifications helps to better elucidate the internal structure of a multi-component material. Especially if the exact constituents of a commercial material, such as the present PLA/CNT/CB/Gr composite, are not precisely known.

For easier visualization, the graphite flakes were colored blue in the images of the cryo-fractured samples. Carbon black and CNTs are not visible at these magnifications (for this, refer to Figure 6). Again, the light microscope images on the right cover the entire cross-section (1.75 mm) of the filaments.

As graphite flakes are highly oriented, the orientation of the particles in the direction of the extrusion flow can be easily observed regardless of the sample preparation and the microscopy method. As the extrusion temperature rises, an optical “increase” in the graphite particle count can be observed. This is caused by the shearing and delamination of the graphite flakes [69,70]. The polymer melt decreases in viscosity, whereby particle structures and agglomerates can be better disintegrated and distributed more homogeneously. This results in improved electrical conductivity. At the highest temperature (220 °C), the particle network appears less aligned along with the extrusion flow, which is potentially a result of incipient structural change in the polymer matrix. Such structural changes can disrupt the conductive pathways between the components of the conductive additives and thus increase the internal resistance.

Thus, the observations and postulated reasons from the electrical conductivity measurements can be supported by imaging methods. However, again, the differences discussed can only be analyzed qualitatively and provide indications, but no data foundation.

### 3.3. Chemical Composition and Thermal Stability

**Figure 8:**
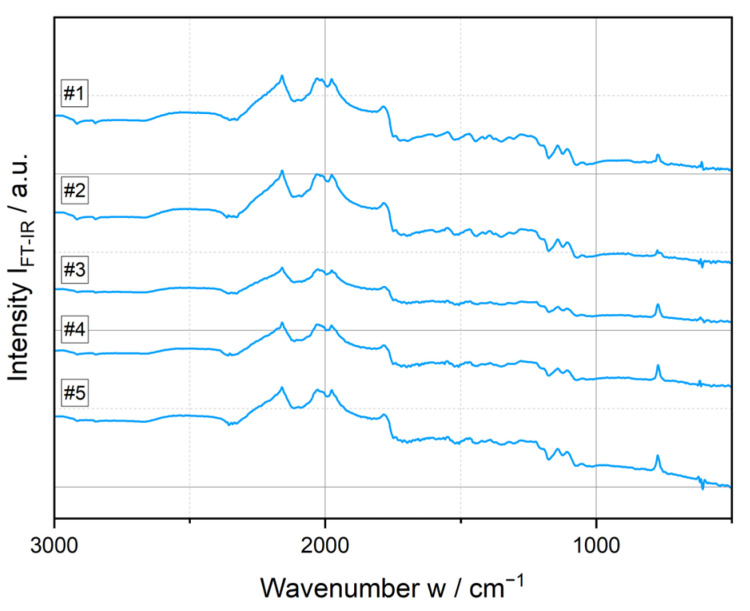
FT-IR spectra for samples of the five parameter sets.

Fourier-transform infrared spectroscopy (FT-IR) and thermogravimetric (TGA) measurements were carried out to exclude inhomogeneities in the particulate composition of the feedstock material and to analyze the potential structural changes observed in Figure 8. Poly(lactic acid) (PLA), like many aliphatic polyesters, is prone to degradation during processing caused by external forces (e.g., mechanical stress, temperature, oxygen, etc.) [71]. A distinction is made between three types of structural changes: thermal (caused by temperature alone), thermo-oxidative (resulting from elevated temperature in an oxygen-containing atmosphere), and thermo-mechanical (when mechanical stress is involved) [72,73]. During Hot Melt Extrusion (HME), all three mechanisms are involved.

The FT-IR spectra do not show any major noticeable differences. Only the peak in the 770 nm^−1^ range, which increases with higher process temperatures, could be an indicator of a change in crystallinity [74].

**Figure 9:**
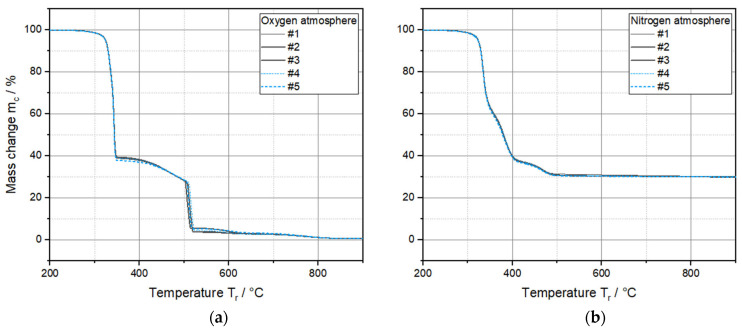
Thermogravimetric analyses of the filament specimens under oxygen (**a**) and nitrogen (**b**) atmosphere.

The thermogravimetric analyses (TGA) were conducted in both oxygen and nitrogen atmospheres. The results of the five parameter sets (Figure 9) differ only marginally. Even during closer examination of individual areas in which large mass losses were detected in a short time, no regularities can be identified. For this reason, the curves are only shown as an overview without magnified areas. The comparison of the two graphs shows that the presence of oxygen leads to faster and more extensive combustion of the material and demonstrates the sensitivity of PLA to thermo-oxidative stresses due to its high diffusivity and reactivity. Signori et al. [71] therefore emphasize the importance of preconditioning the material (drying) and, if possible, the exclusion of oxygen during processing.

The mass contents of the filaments revealed by the results are consistent with the observations of Contreras-Naranjo et al. [61]. The polymer in the samples begins to slowly decompose at approx. 220 °C and leaves a residual mass of just under 39% under an oxygen atmosphere.

The findings of other authors indicate similar temperature thresholds, although the values fluctuate depending on the type of PLA [75,76,77]. Considering that the pure matrix polymer also leaves a residue of a few weight percent at medium temperatures (visible at the 400 °C plateau under nitrogen atmosphere), before it is almost completely decomposed at the maximum temperature of 950 °C, this results in a particulate content of approx. 34 % in weight.

The particulate fillers begin to decompose at approx. 500 °C. This is supported by the studies of Shtein et al. [78]. They carried out comprehensive TGA studies of graphene nanoplatelets (GNPs) and observed that the first combustion of amorphous carbons occurs at 400–500 °C. Between 575 and 750 °C, a second thermal event appears in which the GNPs combust.

### 3.4. Nanomechanical Investigation of the Internal Composite Structure

#### 3.4.1. Methodical Approach of Nanoindentation Mapping and Influence of Particulate Content

The main objective of this work was to perform extensive indentation tests on the PLA/CNT/CB/Gr samples to investigate the internal structure of the solidified polymer. The effects on the electrical conductivity of the composite materials, which so far can only be described qualitatively, are now to be substantiated quantitatively. Micromechanical analyses are barely used in the field of polymer composites, especially for those intended for additive manufacturing. Therefore, the first step is to establish and evaluate a measurement routine.

For this study, the indenter geometry (Berkovich tip) and indentation depths (250 nm) were kept constant. Alongside these parameters, the distance between the individual indents is the most important measurement parameter. Spacings are particularly important for materials where local variations in mechanical properties are the key interest, as in this work, and have been the subject of many studies in recent years [57,58,59,60]. However, as structural material compositions become smaller and more complex, the requirements for progressively finer micro- and nanomechanical surface measurements, hence smaller indentation depths and distances, are also growing. The current standard defines a minimum indent spacing (*d*) of 20 times the indentation depth (*h*) with a Berkovich tip [79]. However, recent studies show that under the same conditions, a *d* to *h* ratio of 1:10 can be applied without the plastic strain zones underneath the indent overlapping [80]. It must also be noted that these standards and suggestions only apply to materials for which indentation tests cause a permanent impression. In the case of materials with perfect elastic recovery, the indents can be lined up as close together as desired.

**Figure 10:**
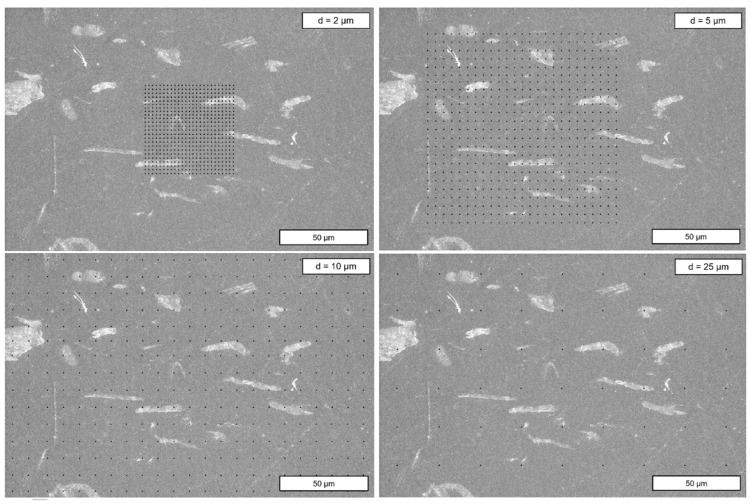
Scale representation of the 25 × 25 arrays showing the examined indent spacings. The exemplary SEM image depicts specimen #3. Each small black triangle represents a single indent. Note that in the case of the 10 and 25 µm indent spacings, only parts of the grid fit in the image as they cover a larger area.

In the scope of this work, indentation spacings of *d* = 2, 5, 10, and 25 µm were applied in preliminary tests. These were arranged in arrays of 25 × 25 points, leading to 625 indents per pattern. Since with each spacing a different area on the surface of the specimen was covered, a schematic visualization of the patterns is presented in Figure 10.

In the course of this work, the reduced modulus of elasticity *E_r_* and the hardness *H* mappings were visualized as contour plots. The color coding of the mappings was divided into 10 areas. *E_r_* values above 10 GPa and *H* values above 0.9 GPa were defined as the upper limit of the color scale in order not to distort it due to particularly high mechanical values caused by graphite flakes or agglomerates exposed on or close to the surface.

**Figure 11:**
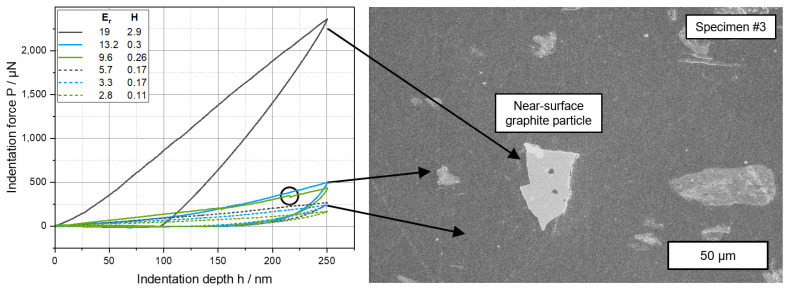
Load–displacement curves of selected data points of sample #3. The circled area shows pop-ins that occur when the material breaks during nanoindentation.

To illustrate this influence, load–displacement curves for a few exemplary *E_r_* and *H* values are plotted in Figure 11. As the indentation process is carried out using displacement control operation mode, the curves become flatter, and the proportion of elastic work decreases. The PLA/CNT/CB/Gr composite combines properties ranging from soft polymer to hard carbon allotropes. The indent resulting in the force-displacement curve, which clearly stands out from the other curves, impacted a graphite particle close to the surface, which was revealed by the polishing process. Similar to graphene, graphite can develop diamond-like mechanical properties [81], which, in addition to the high percentage of elastic deformation work, can be identified by the steep and almost linear gradient of the loading and unloading curve. The following two lines (*E_r_* = 13.2; 9.6 and *H* = 0.3; 0.26) encountered a near-surface particle cluster or a graphite particle covered by a polymer layer. Furthermore, pop-ins can be seen (circled area in Figure 11). They can occur when material structures break during indentation, e.g., through the collapse of loose agglomerates or pores [82]. The flatter the curves become (dashed lines in Figure 11), the more polymer-rich the indentation site is. This softer material possesses only a small amount of elasticity.

**Figure 12:**
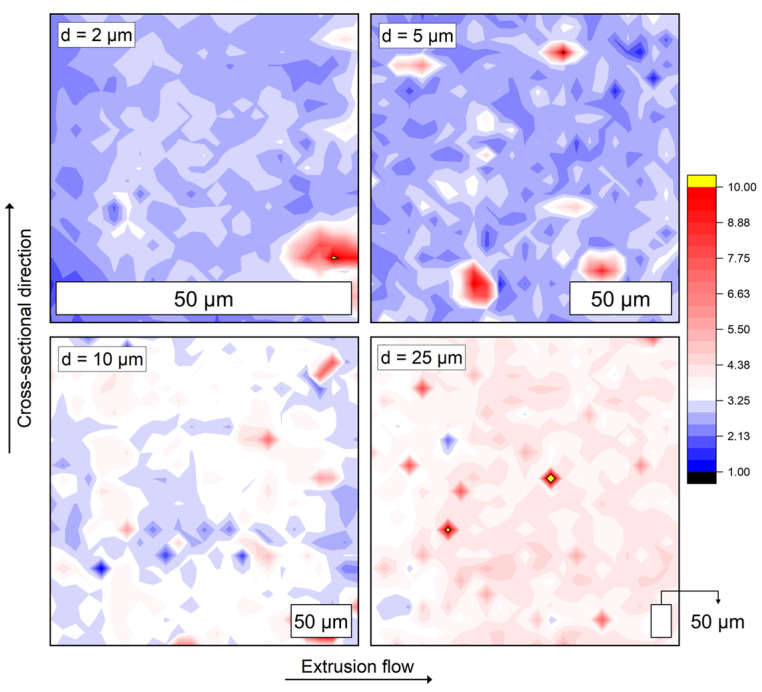
E-modulus mappings for indentation spacings of 2, 5, 10, and 25 µm. The measuring point is located in the concentric filament center of a sample of specimen #1.

The results of the initial spacing tests are plotted in Figure 12 for the *E_r_*. Note that the SEM image in Figure 10 is only a representative measuring area. The mappings for method evaluation were acquired at four different locations. For the two largest distances of *d* = 25 and 10 µm, some measuring points with particularly high (dark red/yellow) and low (dark blue) *E_r_* values can be clearly recognized as structures that stand out from the surrounding area. Here, the indent tip impacts a Gr particle close to the surface. Given Figure 10, it can be concluded that *d* = 25 and 10 µm is too wide. Although a large area can be examined for *d* = 25 µm, the Gr particles fall through the grid due to their elongated shape, which is why this setting is generally unsuitable for the examination of the present PLA/CNT/CB/Gr composite. In the case of *d* = 10 µm, the distance is small enough to study homogeneity effects in the particle distribution, as it can be assumed that most Gr particles are indented at least once. Since this study primarily aims to evaluate the orientation of the particles and the internal structure in the solidified polymer, *d* = 5 and 2 µm have proven to be appropriate.

To achieve the best possible compromise between measuring time, sensitivity, and covered area, for the final testing indent spacings of 5 µm and 25 × 25 arrays were utilized. 625 indents cover a sample area of around 125 × 125 µm. Assuming that the filaments have a round-cylindrical shape with a diameter of approx. 1.75 mm, one row of each pattern covers roughly 7.15% of the aforementioned cross-sectional diameter.

**Figure 13:**
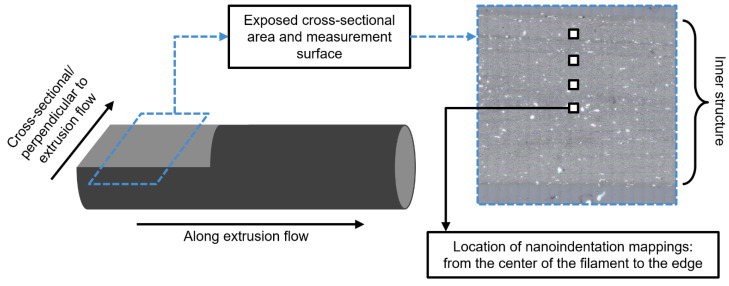
Visualization of the nanoindentation mapping regions across the filament cross-section.

To analyze the microstructural properties across the filament cross-section, the mappings were carried out in different areas. Figure 13 is intended to visualize these regions and the terminology used throughout the course of this work. Furthermore, the results differentiate between particle orientation along the extrusion flow and in the cross-sectional direction.

#### 3.4.2. Influence of the Extrusion Temperature

**Figure 14:**
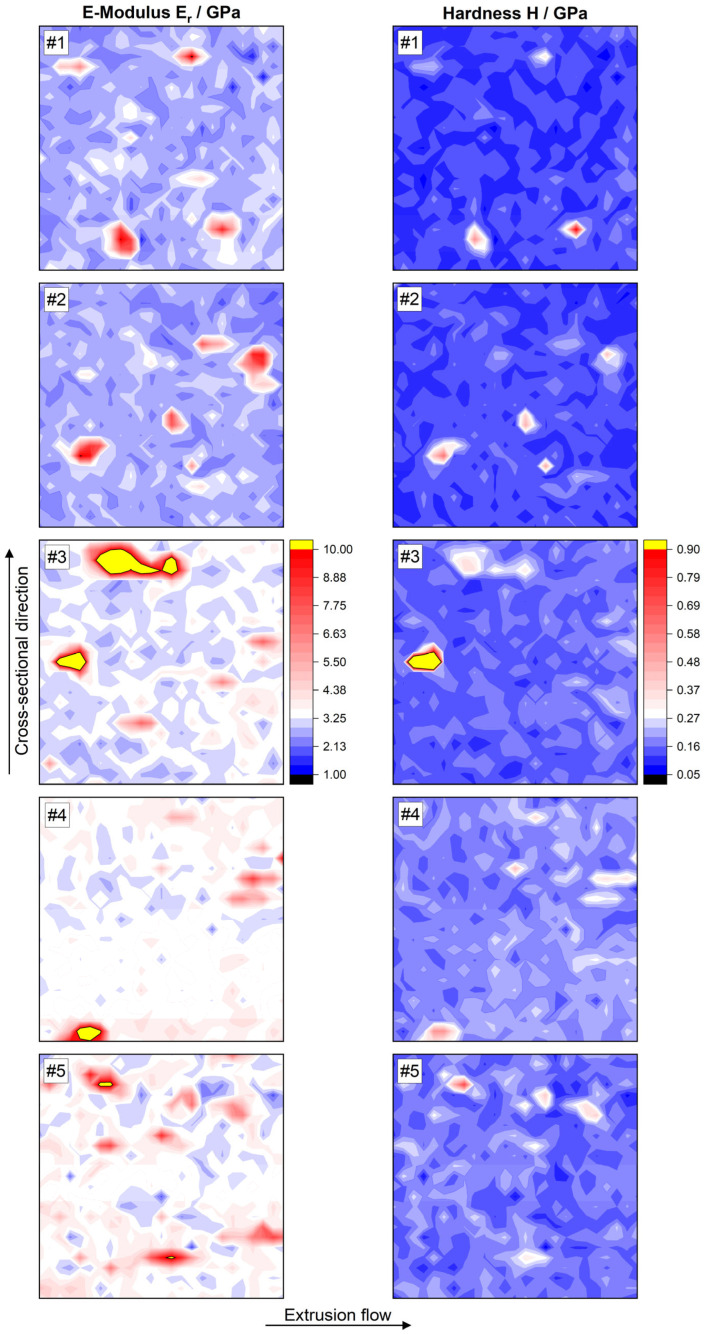
E-modulus and hardness mappings, each in the center of the filament. Indent spacings = 5 µm.

The reduced elastic modulus *E_r_* and hardness *H* mappings are visualized in Figure 14. In each case, the mapping is located in the center of the filament diameter (according to Figure 13). As already shown with optical methods (Figure 7), the orientation of the anisotropic graphite flakes in the direction of extrusion flow can also be clearly observed in the contour plots. Up to 210 °C, the *E_r_* and *H* values increase gradually, which in both cases is reflected in a color shift towards light blue to white. For 220 °C, the values fall and rise equally, but the phenomena are no longer homogeneously distributed across the surface but occur selectively and locally.

Table 3 summarizes the mean *E_r_* and *H* values, including their standard deviations. Sample #5 has a higher mean *E_r_* and a broader distribution of the measured values towards higher *E_r_*, indicating an emerging structural change in the composite material.

As mentioned before, these increasing structural inhomogeneities are most likely caused by structural changes in the matrix polymer due to thermal stress. Cuadri and Martín-Alfonso [83] report that PLA begins to exhibit structural changes at 180 °C. Beginning at normal processing temperatures of approx. 200 °C, thermo-oxidative stress triggers chain scission mechanisms that significantly influence molecular degradation and lead to the formation of degradation products (e.g., linear hydroxyl and carbonyl groups). Gaitanelis et al. [84] used a laser to induce targeted thermal degradation on polyether-ether-ketone (PEEK) samples. Although they observed an increase in E-modulus and hardness of up to 10.8% with short exposure times, the values dropped steadily up to 50% with longer heating durations. They explain this effect as a result of surface carbonization and char layer formation.

However, the matrix material is not solely responsible for these structural changes. The changes observed during processing can most probably be attributed primarily to the incorporation of carbon-based fillers in the formulation. This phenomenon is explained by the high thermal conductivity of these fillers, which are well known for their role in heat-transfer applications [85,86]. Their presence significantly enhances heat distribution within the material, allowing thermal energy to spread more efficiently throughout the polymer matrix during extrusion. Because extrusion involves a relatively small cross-sectional area, heat penetrates the entire profile rapidly, accelerating heat transfer and consequently the melting of the polymer matrix (i.e., reduction in viscosity) compared to systems without such fillers. During this short processing window and given the comparatively small nozzle diameter, the filler particles may shift slightly before the matrix solidifies again. This could result in an apparent increase in particle concentration in certain regions (as discussed in Figure 7).

The micromechanical observations correspond with the measured electrical conductivities (see Figure 5). They increase with growing homogeneity (from sample #1 to #4), as particle clusters and CNTs are better disintegrated and distributed more evenly in the polymer matrix [67]. As a result, the electrical conductivity of samples #3 and #4 is higher. Carbon-based particles have a higher elastic modulus (and therefore a higher hardness) than PLA. Higher extrusion temperatures lead to a lower viscosity of the polymer melt. The improved flow conditions in the screw gap result in better deagglomeration of the carbon black network and increased exfoliation of the graphite flakes. In addition, the CNTs, which are normally present as mechanically entangled solids, are elongated [87]. Consequently, the mechanical values across the entire surface increase.

**Figure 15:**
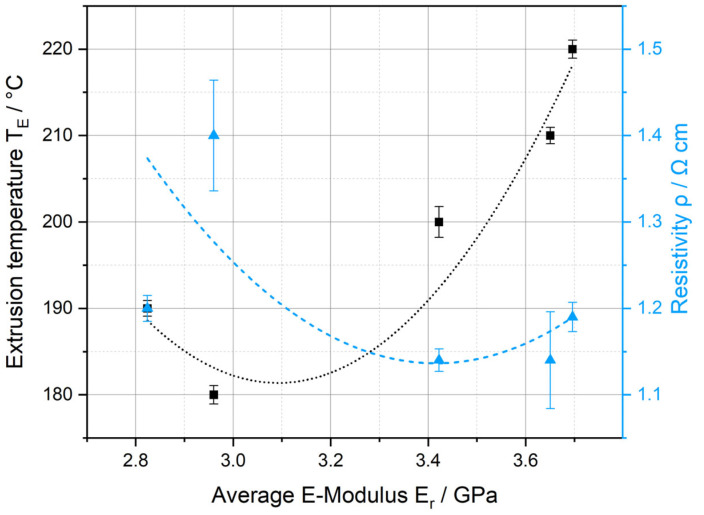
Extrusion temperature and resistivity as a function of the reduced E-modulus.

This provides the process-structure-property relationship of the PLA/CNT/CB/Gr composite shown in Figure 15. The micromechanical properties are determined by the processing temperature in the extruder and, in turn, correlate with the resulting electrical conductivity of the material.

#### 3.4.3. Structural Composition Inside the Filament as a Result of the Hot Melt Extrusion Process

As the E_r_ plots are easier to assess visually due to the broader range of values, the remainder of this study will focus only on the *E_r_* results. 

**Figure 16:**
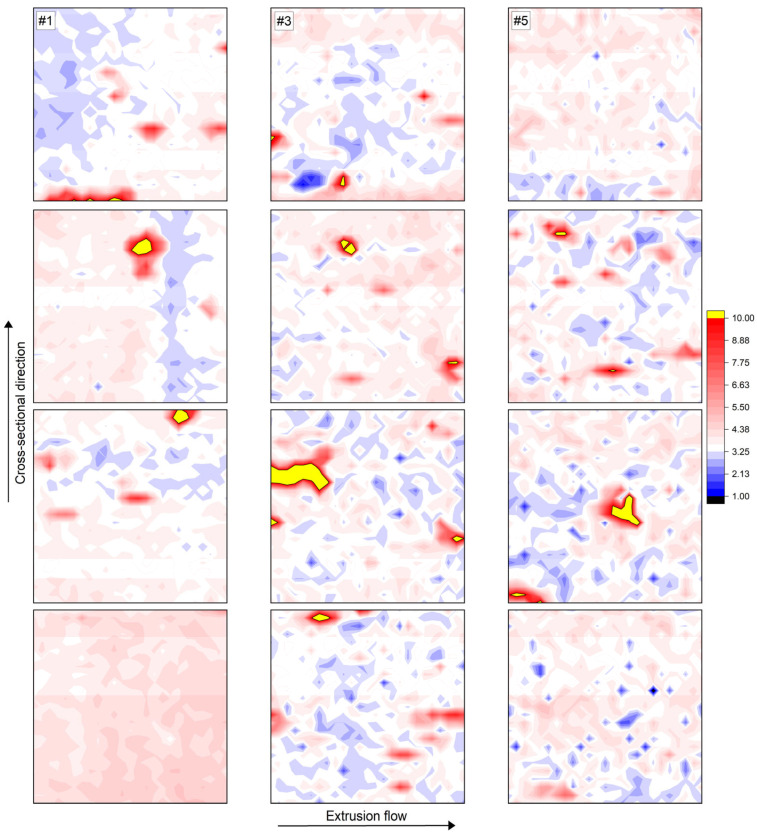
E-modulus mappings of specimens #1, #3, and #5, each from the center (**first row**) of the filament to its edge (**last row**) with equal measuring distances between the grids. Indent spacings = 5 µm.

A homogeneous filament does not have the same composition at every point in the cross-section. Due to the way the Hot Melt Extrusion (HME) process works, as well as the cylindrical concentric shape of the filaments, there are process-related deviations inside the material. For this reason, additional patterns were created for samples #1, #3, and #5, which are positioned in four steps of equal distance from the concentric center of the filament to its edge (according to Figure 13). The *E_r_* plots are summarized in Figure 16. Sample #1 shows significant inhomogeneities, which again explains its poor conductivity as observed in Figure 5. In row two, for instance, a kind of polymer wall can be observed, which may have formed due to the low extrusion temperature of 180 °C. The melt is so highly viscous that particle agglomerates and structures are not disintegrated and swamped by the polymer. Row four appears to be a particle-rich region without much matrix material to surround it. Although the PLA matrix has an electrically insulating effect, it acts as a crucial bridge between the conductive components. Samples #3 and #5 show a more or less homogeneous behavior at all measurement positions, which is consistent with the previous findings regarding particle orientation, structural changes in the polymer, and electrical conductivity.

However, Min Park et al. [88] investigated the orientation of flake-shaped particles in an acrylonitrile butadiene styrene (ABS) melt. Microscopic images showed that the orientation status near the mid-plane differs significantly from that at the edges. They observed particle accumulations in the middle of the sample, which, in the case of this study, represents the concentric center of the filament. To examine this, the average e-moduli over the entire pattern area and their standard deviations are given in Table 4. Apart from the inhomogeneous sample #1, samples #3 and #5 show a slight trend towards decreasing mechanical properties in the direction of the filament edges, which indicates a particle depletion in this area [89]. Nanoindentation patterns of this size do not appear to be sufficient to clearly describe this process-related phenomenon. However, in light microscope images of the entire filament cross-section (Figure 7, right), this effect can be successfully observed.

## 4. Conclusions and Outlook

This study demonstrates the potential of micromechanical analysis to uncover the internal structure of highly filled, anisotropic thermoplastic composites, using a commercial model system consisting of PLA/CNT/CB/Gr. By combining electrical resistivity measurements with nanoindentation and imaging techniques, a direct link between processing parameters—particularly extrusion temperature—and the resulting microstructural and functional properties of the composite is established.

The results show that increasing extrusion temperature improves filler dispersion, reduces agglomeration, and enhances the alignment of anisotropic particles such as graphite flakes and CNTs. This leads to a more homogeneous conductive network and improved mechanical properties, as reflected in both electrical conductivity and local modulus/hardness distributions. However, temperatures above 210 °C induce initial structural alterations of the PLA matrix, which disrupt conductive pathways and introduce local mechanical inhomogeneities.

Nanoindentation mapping revealed distinct spatial variations in stiffness and hardness across the filament cross-section, which correlate with particle orientation and distribution. These micromechanical gradients reflect the complex flow and solidification behavior during extrusion and provide a quantitative basis for understanding the formation of conductive networks. The study has

- established a high-resolution micromechanical mapping method for anisotropic polymer composites;
- demonstrated that even small changes in extrusion temperature significantly affect filler orientation and dispersion;
- revealed local structural gradients and edge effects that are not accessible via global measurements such as electrical resistivity;
- showed that combining nanoindentation with microscopy enables a robust framework for analyzing process-structure-property relationships in conductive polymer systems.

Building on these findings, future work will focus on transferring the developed methodology to custom-formulated composite systems with defined particulate filler types and morphology. By systematically varying process parameters and analyzing their impact on internal structure and particle orientation, a comprehensive database will be established. This will enable the parameterization of simulation models—such as those based on the Finite Element Method (FEM)—to predict functional properties, particularly electrical conductivity, based on known material and process settings. In combination with additive manufacturing, the influence of geometric features on filler alignment and local property distribution will be investigated, supporting the development of design principles for functionally integrated components.

## Figures and Tables

**Table 1:** Temperature profile in the single screw extrusion process.

Specimen Number	Extrusion Temperature TE/°C				
	Zone 4 (Nozzle)	Zone 3	Zone 2	Zone 1	Zone 0 (Feed)
#1	180	135	90	45	22
#2	190	142.5	95	47.5	
#3	200	150	100	50	
#4	210	157.5	105	52.5	
#5	220	165	110	55	

**Table 2:** Assignment of the analytical methods to the sample (preparation) types.

	Analytical Method	Filament Strand	Cryo-Fractured	Resin-Embedded
Chemical	FT-IR	x		
	TGA	x		
Electrical	Electrical resistivity	x		
Optical	SEM		x	x

**Table 3:** Mean *E_r_* and *H* values across the entire pattern area, each in the center of the filament.

Specimen Number	E_r_/GPa	H/GPa
#1	2.99 ± 1.07	0.13 ± 0.05
#2	2.84 ± 0.90	0.14 ± 0.04
#3	3.66 ± 1.79	0.17 ± 0.16
#4	3.69 ± 0.95	0.19 ± 0.04
#5	3.73 ± 1.05	0.17 ± 0.05

**Table 4:** Average reduced E-modulus *E_r_* and standard deviation from the center of the filament to the edge.

Specimen Number	Reduced E-modulus *E_r_*/GPa Location on Filament Cross-Section			
	Center	Middle Center	Middle Edge	Edge
#1	2.99 ± 1.07	3.78 ± 0.98	3.73 ± 0.94	4.30 ± 0.28
#3	3.73 ± 0.92	3.88 ± 1.00	3.98 ± 1.76	3.71 ± 1.09
#5	3.79 ± 0.51	3.73 ± 1.05	3.79 ± 1.36	3.74 ± 0.66

## Data Availability

The original contributions presented in this study are included in the article. Further inquiries can be directed to the corresponding author.

## Abbreviations

The following abbreviations are used in this manuscript:

ABS	Acrylonitrile butadiene styrene	
AM	Additive manufacturing	
CB	Carbon black	
CCVD	Catalytic chemical vapor deposition	
CNC	Cationic cellulose nanocrystals	
CNT	Carbon nanotube	
FEM	Finite Element Method	
FFF	Fused Filament Fabrication	
FGF	Fused Granulate Fabrication	
FT-IR	Fourier-transform infrared spectroscopy	
GNP	Graphene nanoplatelet	
GO	Graphene oxide	
Gr	Graphite	
HME	Hot Melt Extrusion	
MEX	Material Extrusion	
NIR	Near-Infrared	
PEEK	Polyetheretherketone	
PLA	Poly(lactic acid)	
PVDF	Polyvinylidene fluoride	
SD	Standard deviation	
SEM	Scanning electron microscopy	
SSE	Single screw extrusion	
TGA	Thermogravimetric analysis	
US	Ultrasonic	
**Symbols**		
A	Projected contact area	µm^2^
a	Contact distance	mm
d	Indentation spacing	µm
E_r_	Reduced elastic modulus	GPa
P	Indentation force	µN
H	Hardness	GPa
h	Indentation depth	nm
h_f_	Remaining indentation depth	nm
h_max_	Maximum indentation depth	nm
I	Current	A
I_FT-IR_	Intensity	-
m_c_	Mass change	%
P	Indentation load	µN
P_max_	Peak indentation load	µN
R	Resistance	Ω
S	Contact stiffness	N mm^−1^
ß	Geometric constant	-
T_E_	Extrusion temperature	°C
T_r_	Actual temperature	°C
U	Voltage	V
w	Wavenumber	cm^−1^
ρ	Resistivity	Ωcm
